# Continuous Thoracic Sympathetic Ganglion Block in Complex Regional Pain Syndrome Patients with Spinal Cord Stimulation Implantation

**DOI:** 10.1155/2016/5461989

**Published:** 2016-03-29

**Authors:** EungDon Kim, MiSun Roh, SooHyang Kim, DaeHyun Jo

**Affiliations:** Department of Anesthesiology and Pain Medicine, Daejeon St. Mary's Hospital, The Catholic University of Korea, Daeheung-ro 64, Jung-gu, Daejeon 301-723, Republic of Korea

## Abstract

The sympathetic block is widely used for treating neuropathic pain such as complex regional pain syndrome (CRPS). However, single sympathetic block often provides only short-term effect. Moreover, frequent procedures for sympathetic block may increase the risk of complications. The use of epidural route may be limited by concern of infection in case of previous implantation of the spinal cord stimulation (SCS). In contrast, a continuous sympathetic block can be administered without such concerns. The continuous thoracic sympathetic block (TSGB) has been used to treat the ischemic disease and other neuropathic conditions such as postherpetic neuralgia. We administered continuous thoracic sympathetic block using catheter in CRPS patients who underwent SCS implantations and achieved desirable outcomes. We believe a continuous sympathetic block is a considerable option before performing neurolysis or radiofrequency rhizotomy and even after SCS implantation.

## 1. Introduction

Complex regional pain syndrome (CRPS) is an intractable disease caused by a variety of reasons [1]. To treat CRPS, various drugs such as anticonvulsants, tricyclic antidepressants (TCAs), and opioids [1, 2], somatic blocks such as an epidural block, and interventions such as spinal cord stimulation (SCS) are used [3]. However, some patients complain of severe pain even after SCS implantation.

The sympathetic block is widely used for treating neuropathic pain. However, this treatment often provides only short-term relief. Moreover, frequent single sympathetic block may lead to complications such as infections, organ damage, and neural damage. Moreover, complications arising in the thoracic region may be catastrophic.

The continuous thoracic sympathetic block has been used to treat the digital ischemic phenomenon in patients with scleroderma [4]. In addition, its use has been reported in the treatment of neuropathic pains such as postherpetic neuralgia (PHN) and poststroke pain [5].

We report our experience of continuous thoracic sympathetic block using catheter in CRPS patients who had received SCS previously.

## 2. Case Report

*Case  1*.  A 49-year-old female patient suffered from neuropathic pain such as allodynia and hyperalgesia with skin color changes after a right fibular fracture caused by a pedestrian traffic accident that occurred four years earlier. After two years, the pain had spread to her upper limb. One year earlier, after being diagnosed with CRPS by International Association for the Study of Pain (IASP) criteria, the patient received SCS for both upper and lower extremities pain, and the pain subsided to a certain extent. However, she gradually started experiencing pain of numeric rating scale (NRS) 7-8/10 in both upper and both lower extremities.

A stellate ganglion block (SGB), a paravertebral block (PVB), and ketamine infusion were administered to her, but their effects were limited. The patient had positive response from thoracic sympathetic block (TSGB) and the pain reduced by about half or more. However, the effect of this treatment was transient. She complained of allodynia, hyperalgesia, and cold feeling in both hands; further, she complained that the symptoms were more severe in the left part of her body than in the right. We decided to insert a catheter in the left thoracic sympathetic ganglion of the patient to achieve a continuous sympathetic block.

The patient was put in the prone position on a radiology table. A pillow was placed under her precordium to make her vertebral body parallel to the image intensifier of fluoroscopy. The fluoroscopy was rotated in the desired direction by 15–20° and local infiltration was carried out. Thereafter, 18-gauge Tuohy epidural needle was inserted toward the lateral margin of the third thoracic vertebra. After the needle touched the vertebral body of the patient, it was inserted until the needle reached posterior one-third of the vertebral body, in the fluoroscopic lateral view.

By injecting 2-3 mL of a contrast, the location of the thoracic sympathetic ganglion was identified and it was ensured that the contrast did not enter the intravascular, intrapleural, or epidural space. After removing the stylet, a 20-gauge epidural catheter was inserted through an epidural needle. After bolus injection of 6 mL of 2% lidocaine, 0.2% ropivacaine was continuously infused at the rate of 2 mL per hour.

To measure the effect of the sympathetic block, a thermometer was used to measure the temperature of both hands of the patient before and after the procedure. The sympatholysis was considered successful if the temperature difference was 2°C or more [6]. The blood pressure and oxygen saturation of the patient were also monitored.

After the procedure, the pain NRS in the left upper extremity decreased from 8/10 to 3/10. Further, it was observed that the temperatures of the left and right hands increased from 29.2°C to 32.6°C and from 30.1°C to 31.0°C, respectively.

During catheter insertion, the reduction of cold feeling in her left hand was maintained. The patient was taking 40 mg of fast-acting OxyContin per day as a rescue analgesic, but this dosage was decreased to 20 mg after catheter insertion.

A continuous thoracic sympathetic block was administered to the patient for 7 days, and she was then discharged after removing the catheter. After the catheter was removed, the pain in her left upper extremity was still decreased by half, and it was maintained this way for 3 months.

*Case  2*.  A 56-year-old female patient had occipital headache after a rear-end collision that occurred four years earlier. Two years after the accident, pain spread to her whole body. She suffered from allodynia, hyperalgesia, and sudomotor changes. A year earlier, she was diagnosed with CRPS due to IASP criteria and received the SCS procedure for upper and lower extremity pain. Even after the procedure, the patient continued to experience pain of NRS 7-8/10. There was limited effect in the conservative treatment, and her left side had worse symptoms than the right.

The patient also responded to TSGB, but its effect was extremely transient. Catheterization was performed in the left second thoracic sympathetic ganglion of the patient; the procedure and monitoring were conducted in the same way as in case  1.

After the procedure, the pain decreased from NRS 8/10 to 4/10. The temperatures of the left and right hands increased from 31.3°C to 37.1°C and from 32.5°C to 33.4°C, respectively.

During catheter insertion, the pain in the left limb continuously reduced by about half or more. However, the patient complained of discomfort at the catheter insertion site and infuser device made her uncomfortable. Therefore, the catheter was removed after three days. At that time, no signs of infection, hematoma formation, and pneumothorax were observed. The patient reported reduced pain by about half in the left upper extremity for a month after catheter removal.

*Case  3*.  A 56-year-old male patient received SCS implantation three years ago to treat CRPS in his right upper extremity caused by a right wrist fracture that occurred four years earlier. The diagnosis met the criteria for CRPS, recommended by IASP. Despite the SCS implantation, he started complaining of continuous pain with NRS 7/10 after a few months. After a single TSGB, the patient gave positive response to the treatment. However, because the positive effect of the treatment was short-lived, it was determined that catheterization had to be performed in the right second thoracic sympathetic ganglion. The procedure and monitoring were conducted in the same way as in the above two cases.

After the procedure, the pain decreased from NRS 7/10 to 3/10. The temperatures of the right and left hands increased from 29.5°C to 35.9°C and from 30.0°C to 33.5°C, respectively. Further, Horner's sign was observed in right side. Before the procedure, the patient received 50 mg intravenous injection of tramadol for about three times a day as rescue medication. This rescue medication was reduced by 40% during the two weeks of catheter insertion. Moreover, pain remained at a reduced level during this period. After catheter removal, the pain in the right upper limb remained at NRS 4/10 for three months. Thereafter, the patient was able to return to his workplace and resume work normally.

**Figure 1:**
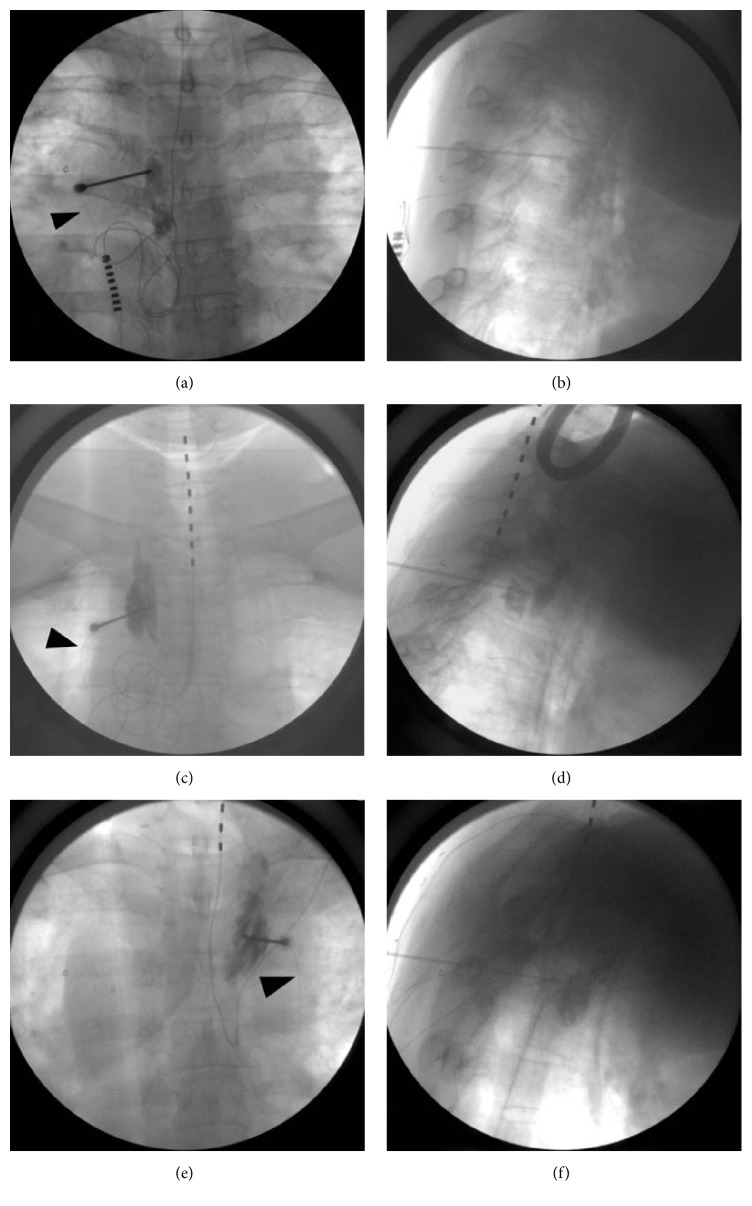
Fluoroscopic images of three cases. (a) and (b) show anteroposterior (AP) and lateral views, respectively, of case  1. (c) and (d) show AP and lateral views, respectively, of case  2. (e) and (f) show AP and lateral views, respectively, of case  3. Black arrowheads indicate the catheter inserted through a Tuohy needle.

In all of the abovementioned three cases, catheterization did not have any effect on the existing SCS. Any procedure related complication such as a pneumothorax was not observed.  Table 1 lists procedure sites, temperature difference between the patients' hands, catheter insertion period, and pain reduction period for the three cases mentioned above. Fluoroscopic views of the operating procedures are shown in Figure 1.

## 3. Discussion

In neuropathic condition, a sympathetic somatic coupling is observed not only in DRG but also in peripheral tissue [7–11]. These observations can explain why the sympathetic block is effective in neuropathic pain.

From the early 20th century, the sympathetic block has been used in CRPS treatment [12], and its effects to various extents have been reported with inconsistent level of evidence [3, 13]. However, in a recent randomized controlled study, TSGB showed short- and long-term positive effects during the treatment of CRPS in upper extremities [14].

A single sympathetic block occasionally has short-term effects. To overcome this drawback, chemical neurolysis or thermal radiofrequency may be performed, but unnecessary complications such as tissue damage or neural damage may occur [15–20].

In a retrospective study in which catheterization was performed in the thoracic sympathetic ganglion using computed tomography (CT) guidance for various neuropathic conditions, the median time required for the severity of symptoms reducing to half was 216 hours [5]. This implies that the effect duration of the single sympathetic block may be insufficient to provide relief from neuropathic symptoms in all cases. When a single sympathetic block was not effective, the patient can be assumed to have sympathetically independent pain (SIP). However, continuous blocking of sympathetic outflow can be effective.

Recently, SCS implantation has emerged as the treatment method for CRPS [1, 3]. However, intractable pain even after SCS implantation is occasionally the outcome. In cases when SCS has been already conducted and a block must be administered for pain control, the use of epidural route can be limited by risk of infection around stimulator lead when the target nerve of epidural block is adjacent to the stimulator lead that is already inserted in the epidural space. Moreover, continuous epidural infusion performed using a catheter may increase this risk even more. In contrast, a continuous sympathetic block is remote from the SCS stimulator leads and can be administered without such risk. To our knowledge, the current cases are the first ones that involve a continuous sympathetic block in patients with CRPS who have received SCS.

In the abovementioned cases, the pain reduction lasted only for a few months after the administering of a continuous sympathetic block. However, this duration was significantly longer than that achieved by administering a single block. We maintained the injection of local anesthetics at 2 mL per hour. However, this rate is relatively slow compared to previous studies on the continuous thoracic sympathetic block in which 0.32% mepivacaine and 0.2% ropivacaine were infused at 4 and 10 mL per hour, respectively [4, 5]. If adequate concentrations and infusion rates are employed, better clinical outcomes may be expected.

A study reported that a better clinical outcome was obtained when TSGB was administered within a year after the occurrence of pain associated with various neuropathic conditions [21]. We believe a continuous sympathetic block can be effective in blocking disease progress in the early stages of neuropathic pain and can avoid motor and sensory deficit that may be caused when an epidural block is administered. Therefore, a continuous sympathetic block can minimize the interruption of the rehabilitation treatment.

In conclusion, a continuous thoracic sympathetic ganglion block using a catheter showed desirable outcomes in patients with CRPS who had received SCS. In the treatment of neuropathic pain conditions, we believe that even if a single sympathetic block was not effective, a continuous sympathetic block using a catheter should be considered before performing a procedure that may result in permanent tissue or nerve injury, such as chemical neurolysis or the thermal radiofrequency rhizotomy.

## Figures and Tables

**Table 1:** Directions and levels of the procedure, temperature difference, and durations of catheterization and pain reduction.

	Site of procedure	NRS before procedure	NRS after procedure	Temperature difference	Duration of catheterization	Duration of pain reduction
Case 1	T3, left	8	3	2.5°C	7 days	3 months
Case 2	T2, left	8	4	3.9°C	3 days	1 month
Case 3	T2, right	7	3	2.9°C	14 days	3 months

## Additional Points

Thoracic sympathetic block (TSGB) is a useful treatment method for management of neuropathic pain of upper extremities. But single TSGB often provides only temporary effect. Besides, frequent procedures in the thoracic region may cause severe complications. In case of spinal cord stimulation (SCS) implanted previously, the use of epidural route for pain control can be limited. In case of complex regional pain syndrome of upper extremities with previous SCS implantation, continuous TSGB showed desirable results. We believe a continuous TSGB is a reasonable option for such a condition.
